# Geckos as Springs: Mechanics Explain Across-Species Scaling of Adhesion

**DOI:** 10.1371/journal.pone.0134604

**Published:** 2015-09-02

**Authors:** Casey A. Gilman, Michael J. Imburgia, Michael D. Bartlett, Daniel R. King, Alfred J. Crosby, Duncan J. Irschick

**Affiliations:** 1 Graduate Program in Organismic and Evolutionary Biology, University of Massachusetts Amherst, Amherst, MA, 01003, United States of America; 2 Polymer Science and Engineering Department, University of Massachusetts Amherst, Amherst, MA, 01003, United States of America; 3 Department of Biology, University of Massachusetts at Amherst, Amherst, MA, 01003, United States of America; Duke University Marine Laboratory, UNITED STATES

## Abstract

One of the central controversies regarding the evolution of adhesion concerns how adhesive force scales as animals change in size, either among or within species. A widely held view is that as animals become larger, the primary mechanism that enables them to climb is increasing pad area. However, prior studies show that much of the variation in maximum adhesive force remains unexplained, even when area is accounted for. We tested the hypothesis that maximum adhesive force among pad-bearing gecko species is not solely dictated by toepad area, but also depends on the ratio of toepad area to gecko adhesive system compliance in the loading direction, where compliance (*C*) is the change in extension (Δ) relative to a change in force (*F*) while loading a gecko’s adhesive system (*C* = dΔ/d*F*). Geckos are well-known for their ability to climb on a range of vertical and overhanging surfaces, and range in mass from several grams to over 300 grams, yet little is understood of the factors that enable adhesion to scale with body size. We examined the maximum adhesive force of six gecko species that vary in body size (~2–100 g). We also examined changes between juveniles and adults within a single species (*Phelsuma grandis*). We found that maximum adhesive force and toepad area increased with increasing gecko size, and that as gecko species become larger, their adhesive systems become significantly less compliant. Additionally, our hypothesis was supported, as the best predictor of maximum adhesive force was not toepad area or compliance alone, but the ratio of toepad area to compliance. We verified this result using a synthetic “model gecko” system comprised of synthetic adhesive pads attached to a glass substrate and a synthetic tendon (mechanical spring) of finite stiffness. Our data indicate that increases in toepad area as geckos become larger cannot fully account for increased adhesive abilities, and decreased compliance must be included to explain the scaling of adhesion in animals with dry adhesion systems.

## Introduction

The ability of animals such as lizards and invertebrates to climb using specialized climbing structures has been a focus of research for many decades [1–3]. Adhesion in animals has evolved several times, and includes various morphological specializations for both “wet” and “dry” adhesion [4–5], although we note that the mechanisms that underlie wet and dry adhesion are not necessarily mutually exclusive. Wet adhesion has evolved in mussels, frogs, mammals, and some flies, in which each secrete oily or mucus-like substances to create an adhesive bond with the surface [3,6]. Other species (e.g., insects, spiders, and geckos) have evolved various kinds of dry adhesive systems, which rely on the creation of intimate van der Waals bonds with substrates via microscopic setae, typically found on the toes of animals that climb, or that seize prey (e.g., spiders, kissing bugs) [7–8]. These setae are hair-like epidermal projections that conform even to rough surfaces (e.g., rock) [9–16].

One of the central unresolved issues regarding the evolution of adhesion concerns how adhesive force scales as animals change in size. There is substantial variation in body size among different species that employ both dry and wet adhesion. For example, gecko species with toepads vary in body size from about 2 g to over 300 g, representing a 150x scaling in body mass [17]. The classical view is that as body size increases, the size of the climbing structures also increases, and therefore the total maximum adhesive force should increase with pad area. However, although both maximum adhesive force and toepad area increase with body size both within and among pad-bearing lizards (geckos and anoles, [1,15,17–19]), there remains significant variation (close to 50%) in maximum adhesive force that is unexplained. As an example of a scaling study, Webster and colleagues [15] showed that pad area found isometric scaling with body mass, and setal density, so that spacing, and diameter remained relatively constant among different sizes across ontogeny within the gecko *Chondrodactylus bibronii*. We note that climbing in geckos is complex [16,20], and measurements of maximum adhesive force represent one aspect of their adhesive ability which we discuss more fully below. For species with setae (insects, lizards), one of the main theories for how larger species compensate for this variability is by increasing density [21–22]. Nevertheless, investigations of setal density among groups of climbing lizards show that it does not change with size [15,23]. Therefore, other factors besides pad area must be responsible for the upscaling of adhesion.

Here, we test a different and largely ignored mechanism, namely that changes in the compliance of the adhesive system explain the scaling of maximum adhesive force, where compliance (*C*) is the change in extension (Δ) relative to a change in force (*F*) during the loading of a gecko’s adhesive system (*C* = dΔ/d*F*). While stiffness describes a systems resistance to deformation, compliance is the inverse of stiffness, where a low compliance is equivalent to a high stiffness. Specifically, we test the hypothesis that decreases in compliance with increased animal size explain much of the increase in maximum adhesive force that enables larger animals to climb. We test this hypothesis with several species of gecko that vary dramatically in size. This hypothesis is based on recent work [24–26] which has shown, using synthetic gecko-like adhesives, that decreased compliance enables stronger adhesion. Specifically, Bartlett et al. [25] provide a scaling equation which shows that reversible maximum adhesive force in synthetic and biological adhesive systems can be explained largely through a ratio of the adhesive contact area to the compliance in the direction of loading. In other words, in order for maximum adhesive force to increase for a constant or conserved contact area, the entire adhesive system must become stiffer in the direction of loading [25,27].

The ratio of toepad area to system compliance is a non-intuitive scaling parameter for gecko adhesion. However, its role can be described by straightforward physical principles applied within the constraints of biological locomotion. Let us imagine that the gecko adhesive system is comprised of a simple pad in which integrated units (e.g., tendon, skin, connective tissue, setae) represent a series of elastic springs that connect the pad to the load-bearing skeleton [20,28]. When the gecko pad is attached to a substrate and is adhering to a surface, the force applied to the substrate is equal to *F* = (1/*C*)*x*, or the product of the stretch (*x*) in the springs, pad, and substrate, and the system stiffness (or the inverse of system compliance (1/*C*). Assuming that, (1) the system is in equilibrium prior to slipping or losing attachment, (2) the loss of attachment or slipping is sudden, and (3) the energy in the system is elastically conserved, then the maximum force sustained prior to detachment occurs when the energy in the system equals the interfacial energy of the pad/substrate contact area (*A*), or ½(1/*C*)*x* ^2^ = *G*_c_ *A*, where *G*_*c*_ is the energy per unit area defined by the nature of the bonds at the interface [25,26]. Since the system is assumed to be elastic, the stretch at detachment (*x*_c_) is equal to *CF*_c_, where *F*_c_ is the force capacity at attachment, and thus, *F*_c_ ∝ √G_c_ $\surd \frac{A}{C}$. For geckos, *G*_c_ is governed by van der Waals forces, which do not scale with body size. Therefore, the adhesive cling force scaling parameter is $\surd \frac{A}{C}$.

The implications of this proposed relationship for living organisms is profound. This hypothesis predicts that when one compares climbing geckos of differing sizes, the adhesive system of larger geckos should be substantially stiffer than for smaller geckos, assuming approximately constant ratios of toepad area to body mass. As noted in other work [1,15], there is no strong evidence for a strong departure from isometric expectations for toepad area. Stiffness in geckos is controlled by many factors, such as the size of the adhering structure, the muscles, and the arrangement of soft tissues within the pad and limb. For example, geckos possess a unique arrangement of tendons in their toepads, which play a vital role for maintaining stiffness during adhesion [20,25]. Gecko species are divergent in their tendon structure [5,28], Kuo et al., unpub. data), which seem to include differences in the overall shape, size, and placement of the tendons within the foot, although these differences need to be quantified. Given the importance of tendon foot anatomy for influencing adhesion in geckos [25], this suggests there may exist differences among species in the compliance of the adhesive system.

Although this relationship between maximum adhesive force and compliance has been proposed [25], it was only tested on either synthetic adhesives, or on suborganismal features (e.g., setae), and only one whole living organism was tested (*Gekko gecko*). Therefore, the role of compliance in how maximum adhesive force is scaled among different gecko species is still unknown. To test the hypothesis that maximum adhesive force is best predicted by the ratio of area to compliance (*A/C*), we quantified maximum adhesive force, toepad area, and system compliance for gecko species that vary dramatically in body size. Geckos are a good choice for testing this hypothesis because they vary greatly in size, and there is a large body of work on the anatomy and function of gecko toepads (e.g., 1,10,16,20], as well as synthetic gecko-like adhesives [1,15,25,29–33].

We chose a range of six gecko species that include at least one smaller size class (juveniles) within one of the species (*Phelsuma grandis*) to expand the size range we investigated. We chose these species for several reasons. First, they represent a range of phylogenetically divergent gecko species [5] belonging to several genera (e.g., *Gekko*, *Phelsuma*, *Gehyra*), thus allowing us to test the generality of our results across a broad spectrum of species. Second, these species represent a wide range of body sizes, from about 2 g (gold-dust geckos, *P*. *laticauda*) to almost 100 g (tokay geckos, *Gekko gecko*). Since our primary goal was examination of size in relation to toepad area, maximum adhesive force, and compliance, having a wide size range was critical. Third, these species were readily available in the commercial pet trade, adjust well to captivity, and performed well in adhesion tests. In addition to conducting adhesion tests on live animals, we also demonstrated the mechanistic relationship between adhesive area and *A/C* by performing adhesion measurements with synthetic materials that vary in compliance. We performed experiments on both single synthetic pads and on pads that more closely resemble a five-toed gecko foot, and we compare these results to that for live geckos.

## Methods

### Ethics statement

This work was carried out under an Institutional Animal Care and Use (IACUC) protocol 2012–0064 from the University of Massachusetts at Amherst to D. Irschick. All geckos were obtained from legal vendors through this protocol. All experiments in this paper are approved in this protocol.

### Experiments with live geckos

**Fig 1:**
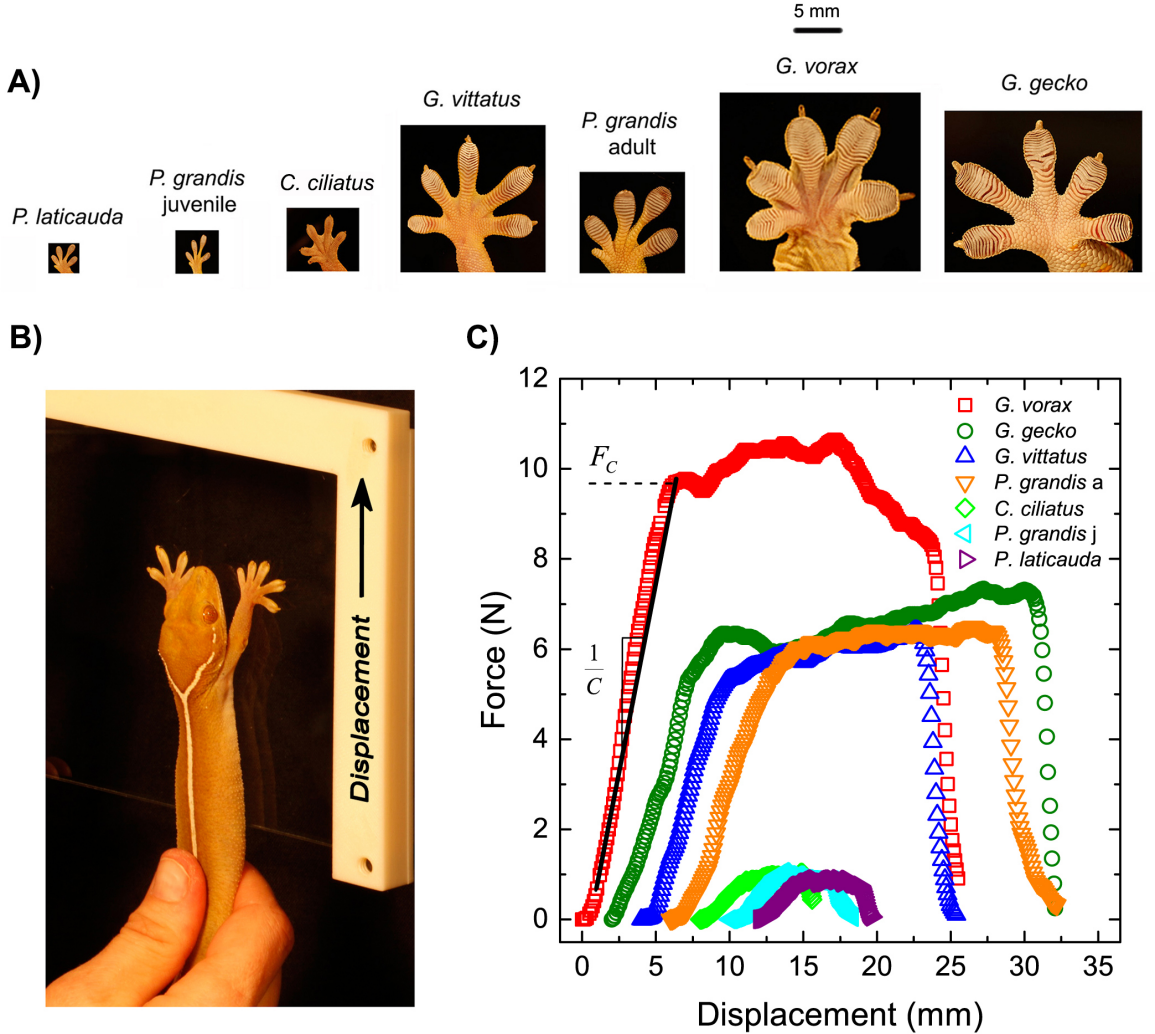
A) Images of one front foot of each species used in this study, displayed in proportion to one another, with species ordered by increasing body size. B) Experimental method used to record maximum adhesive force (*F*_*c*_) and system compliance (*C*), showing *G*. *vittatus* with front feet placed flat on glass plate, just before plate extends upward (denoted by the arrow) away from gecko, and C) sample recordings from each species showing *F*_*c*_ and gecko stiffness (1/*C*).

We measured maximum adhesive force and system compliance in six species of gecko (see Table 1, Fig 1A). We included juvenile and adult *P*. *grandis* as separate groups to bolster our sample sizes at the low end of the animal size spectrum. Geckos were maintained individually in either plastic aquaria (42.9x15.2x21.6 cm) or 10-ga glass aquaria supplied with coconut coir mulch and a wood basking perch and bark chips. The cages were sprayed with water daily, and the lizards were fed calcium-dusted crickets three times a week and provided with a 12h:12h light:dark cycle using an aluminum clamp work light and a 65W incandescent bulb.

**Table 1:** Body size and toepad area of both front feet of the species used in this study. Traits are shown as mean ± 1 s.e.m., with the range in parentheses.

Species	N	Mass (g)	Snout-vent length (mm)	Front Toepad Area (mm^2^)
*Phelsuma laticauda*	3	2.5 ± 0.1 (2.3–2.6)	46.0 ± 1.0 (44.0–47.0)	13.4 ± 0.8 (12.1–14.8)
*Phelsuma grandis* juvenile	5	3.1 ± 0.3 (2.5–3.8)	51.2 ± 1.5 (46.0–55.0)	21.5 ±1.4 (17.9–26.1)
*Correlophus ciliatus*	3	7.7 ±0.3 (7.0–8.0)	68.7 ± 0.9 (67.0–70.0)	43.9 ± 1.9 (43.3–47.5)
*Gekko vittatus*	8	22.1 ± 2.2 (15.0–30.0)	112.1 ± 6.0 (95.0–130.0)	151.9 ± 15.2 (95.2–209.8)
*P*. *grandis* adult	5	30.3 ± 2.7 (21.0–36.0)	97.0 ± 2.0 (90.0–101.0)	101.4 ± 4.1 (88.0–110.0)
*Gehyra vorax*	4	43.3 ± 3.4 (36.0–50.0)	125.8 ± 5.3 (110.0–133.0)	130.9 ± 12.2 (95.8–152.5)
*Gekko gecko*	5	51.6 ± 12.7 (27.0–97.0)	130.0 ± 6.0 (116.0–150.0)	195.3 ± 18.5 (125.2–233.2)

After the maximum adhesive force trials (see below), we measured mass, snout-vent length (SVL), and combined toepad area of both front feet for each gecko. We measured toepad area of each gecko by having the gecko cling to a vertical glass plate and we captured an image with a Canon EOS 5D (Canon U.S.A., Inc.). We then measured the area of each toepad using Image J (Rasband, 1997–2009) and combined all toepad areas to calculate the total toepad area for the front feet of each individual. Prior studies show that this method is effective for measuring toepad area [19].

We measured maximum adhesive force (*F*_*c*_) and system compliance (*C*) under shear loading on glass using an Instron 5500R tensile testing instrument (Instron Worldwide, Norwood, MA., USA). The glass was held vertically in place by a custom built holder, which was rigidly attached to the crosshead of the Instron. Geckos were not used for adhesion trials if they were actively shedding, as shedding might reduce adhesive performance. Further, because the geckos were pulled gently, we did not notice any cases of setae being lost or toepad damage. We performed force capacity measurements by gently holding the animal’s torso in place with one hand and encouraging the placement of both front feet onto the glass, which was then displaced at velocities of 300 mm/min for the larger geckos (> 70 mm SVL), or 150 mm/min for the smaller geckos (≤ 70 mm SVL) (Fig 1B). The gecko-handler (Casey Gilman) kept her arm and hand as still as possible to avoid any additional contribution to the force measurements. We opted to run the geckos of different sizes at different speeds because a faster speed will be much more challenging for a small (~2 g) gecko to adhere to glass compared to a large gecko. We performed an additional experiment with a larger gecko, adult *Gehyra vorax* (N = 8), in which we examined the effects of rate (150 mm/min or 300 mm/min) by alternately varying rates, and then measuring their force capacities and compliance. When we compared the original data (using the two rates as described above and below) to the new data that includes 150 mm/min for the *Gehyra vorax*, the slope of the line between A/C (x-axis) versus maximum adhesive force (y-axis) did not differ significantly (t-test, t = 0.51, P>0.50). This indicates that variations in rate did not greatly affect our overall conclusions.

We measured maximum adhesive force several times (between 5–9 times per gecko) to obtain a maximum effort for adhesion. A successful trial consisted of the lizard placing both front feet on the substrate and completely extending its forearms. We considered extension of the forearms to be important because we observed that when lizards did not extend their arms, they often did not place all toepads flush against the substrate. After maximum force was achieved, the gecko toe pads would begin to slip, which can be seen as a plateau or decrease in force, and then we would reset and repeat the test (Fig 1C). Measurements were recorded in either one afternoon or on two afternoons at least one week apart to allow the geckos to recover between trial days. Although the species vary in whether they are diurnal (*Phelsuma*) or nocturnal (the other species), past work in the Irschick laboratory over the past 20 years has shown little evidence of notable differences in adhesive effort at different times of day (Irschick, pers. obs.) and thus we opted to proceed with a standardized time rather than try and use different times (e.g., day and night). The temperature of the room in which the force measurements were recorded was 20 ± 1.0°C. The maximum force of adhesion does not differ with temperature in geckos [34].

To determine the values for *C* and force capacity in a systematic manner for all gecko and synthetic adhesive measurements, a two point analysis method was adopted. The two data points correspond to transitions in the shear adhesion measurements. The first datum for the gecko measurements is the point at which a linear relationship begins, corresponding qualitatively with the instance when the specimen’s forearms were completely extended. For synthetic adhesive measurements, the first datum is the initial minimum force before loading the adhesive. The second datum is the initial drop in force, corresponding to the onset of slip between the specimen toepad or adhesive pad and the glass substrate. The force for this second datum is the maximum adhesive force capacity. A least squares linear fit was applied to the data between the first and second data points. The slope of the least squares fit is the system stiffness, from which the system compliance (*C*) is calculated as the inverse. Detailed representative plots of datum selection are included in the supplemental information (S1 Fig).

### Experiments on a synthetic “model gecko” system

We constructed “model gecko” systems, in which a synthetic adhesive pad is attached to a glass substrate and a synthetic tendon (mechanical spring) of finite stiffness is placed in series with the adhesive pad. We simulated the dynamic motion of climbing by loading the structure through a controlled displacement far from the interface and applied displacement, then recorded force until the pad detached from the substrate. The test was then rerun using a synthetic tendon with a different compliance without changing the adhesive pad or glass substrate. We fabricated synthetic adhesives by filling clean glass molds with oligomeric polydimethylsiloxane (PDMS) and a curing agent (Dow Corning Sylgard 184) with a 10:1 ratio by mass. We placed plain weave nylon fabric (Jo-Ann Fabric and Crafts) on top of the uncured PDMS to impregnate the fabric and we then allowed the adhesives to cure at room temperature for at least three days. We then cut the adhesives to a size of 20 mm x 15 mm x 1mm (width, height, thickness) for a contact area *A* = 300 mm^2^. The synthetic adhesives were applied by hand to a clean glass substrate and were tested in a lap shear geometry at a displacement rate of 10 mm/min. This test was performed 9 times and each test was concluded when the adhesive separated from the glass. The synthetic adhesive was then connected in series to a spring of one of three levels of compliance and the lap shear adhesion experiments were repeated. Here we report the mean of the 9 trials for each level of compliance.

### Statistical Analyses

We used the maximum value of all successful trials for each individual as the measure of maximum adhesive force for each lizard, and used the averaged compliance estimates for all successful trials as the system compliance value. We excluded eight lizards from the analyses (generally one or two per species) because they did not perform well during the trials (i.e., would not adhere to the glass).

We used mass as our estimate of size in lizards and included snout-vent length as a comparison, as it is often used as an index of body size in reptile literature [35]. The following variables and abbreviations are used throughout: Maximum adhesive force (*F*_*c*_), Snout-vent length (SVL), Toepad area (*A*), System compliance (*C*). We first used generalized linear models to determine the following relationships (all variables log-transformed, first variable is dependent variable, second variable is independent variable): (i) *F*_*c*_ vs SVL; (ii) *A* vs SVL; (iii) *F*_*c*_ vs *A*; (iv) *C* vs. SVL; (v) *F*_*c*_ vs *C*; (vi) *F*_*c*_ vs *A*/*C*.

We investigated whether toepad area (*A*), compliance (*C*), or the ratio of toepad area to compliance ratio (*A/C*) best explained maximum adhesive force across our gecko species. To do this, we compared the resulting outputs of the above models to determine which variable(s) exhibited a significant relationship to maximum adhesive force with the smallest amount of unexplained variation. We also examined the relationships between variables within *P*. *grandis* and compared the resulting linear models to those that included all the species using t-tests to determine whether ontogenetic relationships matched those across species. To test for differences in maximum adhesive force across species, we conducted a one-way ANOVA using maximum adhesive force divided by toepad area (i.e., Irschick et al. 2006, Stress, N/mm^2^, also known as average nominal stress), followed by post-hoc Tukey’s HSD pair-wise comparisons between species. To account for multiple statistical analyses, we used a sequential Bonferroni test for all tests used in this study.

## Results

**Fig 2:**
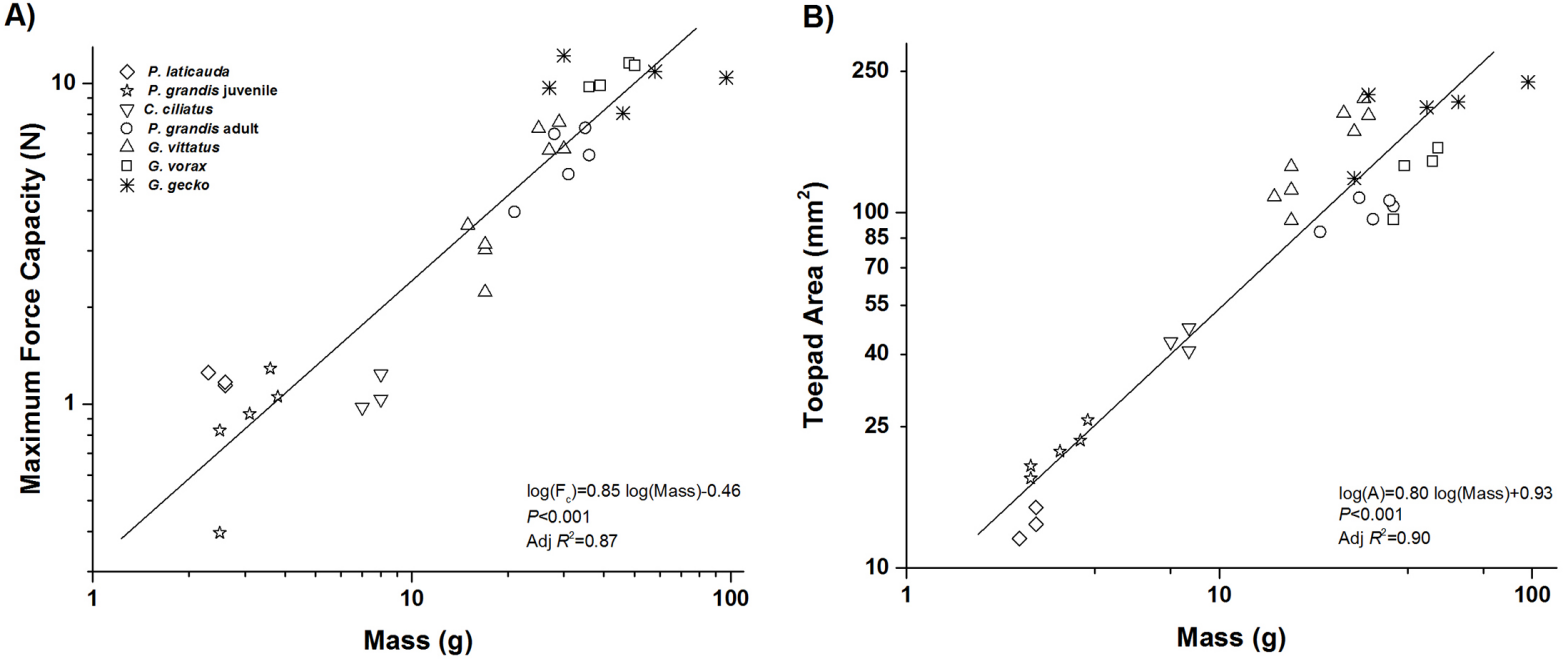
Relationship between gecko size (mass), maximum adhesive force, and adhesive area (toepad size). A) As expected, a gecko’s maximum adhesive force significantly increases with increasing animal size. B) This increase in maximum adhesive force with animal size is partly due to the significant positive relationship between toepad size and body size in geckos.

As expected, we found that as geckos become larger, their toepads also become larger and their total maximum adhesive force increased. There was a significant and positive relationship between body mass and maximum adhesive force (log(*F*_*c*_) = 0.85log(Mass)– 0.46, *P*<0.001, Adj. *R* ^2^ = 0.87, Fig 2A), and mass and toepad area in geckos (log(*A*) = 0.80log(Mass) + 0.93, *P*<0.001, Adj. *R* ^2^ = 0.90, Fig 2B). However, increases in mass alone, which is tightly correlated with toepad area, did not fully explain increases in maximum adhesive force (Adj. *R* ^2^ = 0.87, and thus remaining unexplained variability ~13%). SVL scaled to maximum adhesive force (log(*F*_*c*_) = 2.40log(SVL)– 4.14, *P*<0.001, Adj. *R* ^2^ = 0.86) and toepad area (log(*A*) = 2.36log(SVL)– 2.71, *P*<0.001, Adj. *R* ^2^ = 0.96) similarly as mass in geckos.

**Fig 3:**
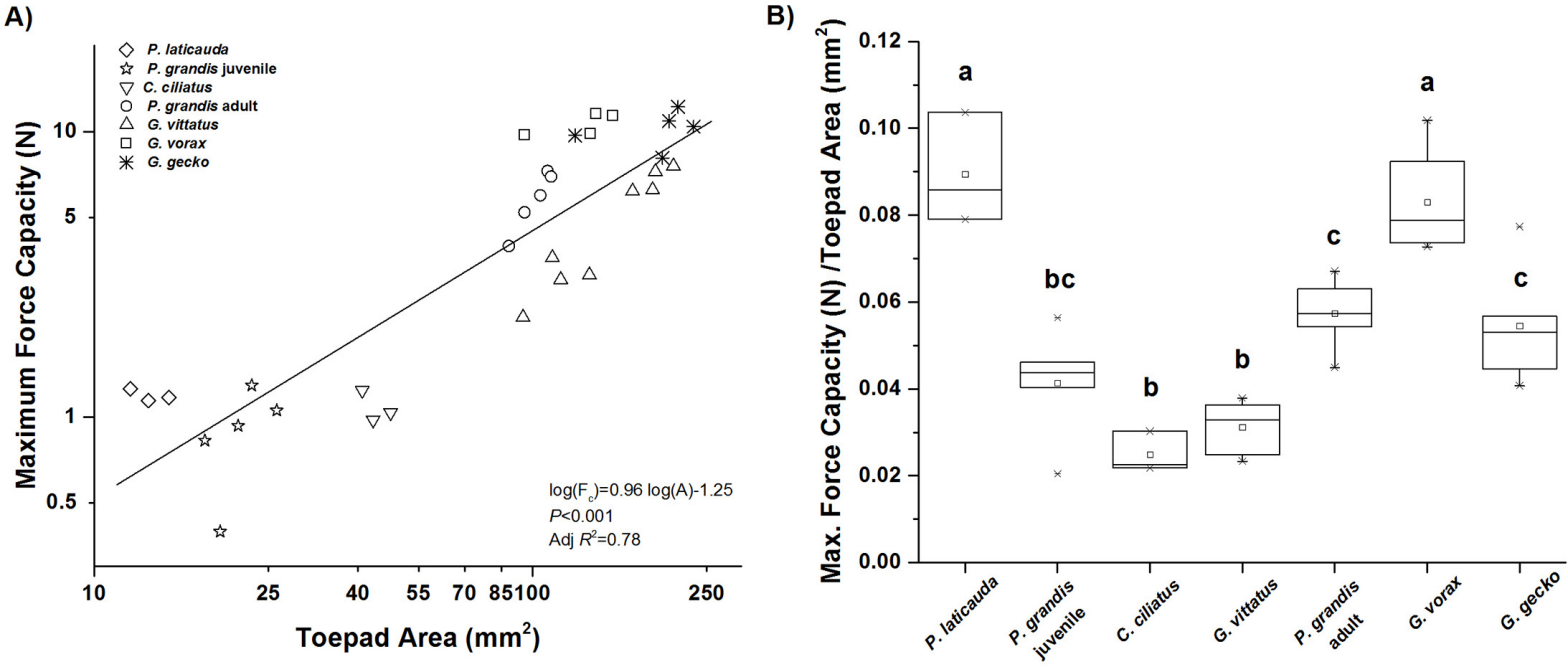
Relationship between animal size and maximum adhesive force. A) Although toepad area significantly increases with animal size (as seen in Fig 2), this relationship only weakly explains the increase in maximum adhesive force associated with increases in animal size. B) Toepad size-adjusted maximum adhesive force (stress) for each species tested. Species are ordered by increasing body size. Boxes show minimum, maximum and median values for each species. Letters above boxes denote significant differences between species (*P* ≤ 0.01).

Maximum adhesive force increased significantly with increases in toepad area (log(*F*_*c*_) = 0.96log(*A*)– 1.25, *P*<0.001, Adj. *R* ^2^ = 0.78, Fig 3A). However, this relationship only partially explains the increase in maximum adhesive force associated with increases in body size, with about 22% of variation unexplained. The unexplained variability in adhesive force, even when the effects of toepad area are accounted for can be seen in Fig 3B. There were significant differences in maximum adhesive force values among all species (*P*<0.001) and between species pairs (*P*≤0.01).

**Fig 4:**
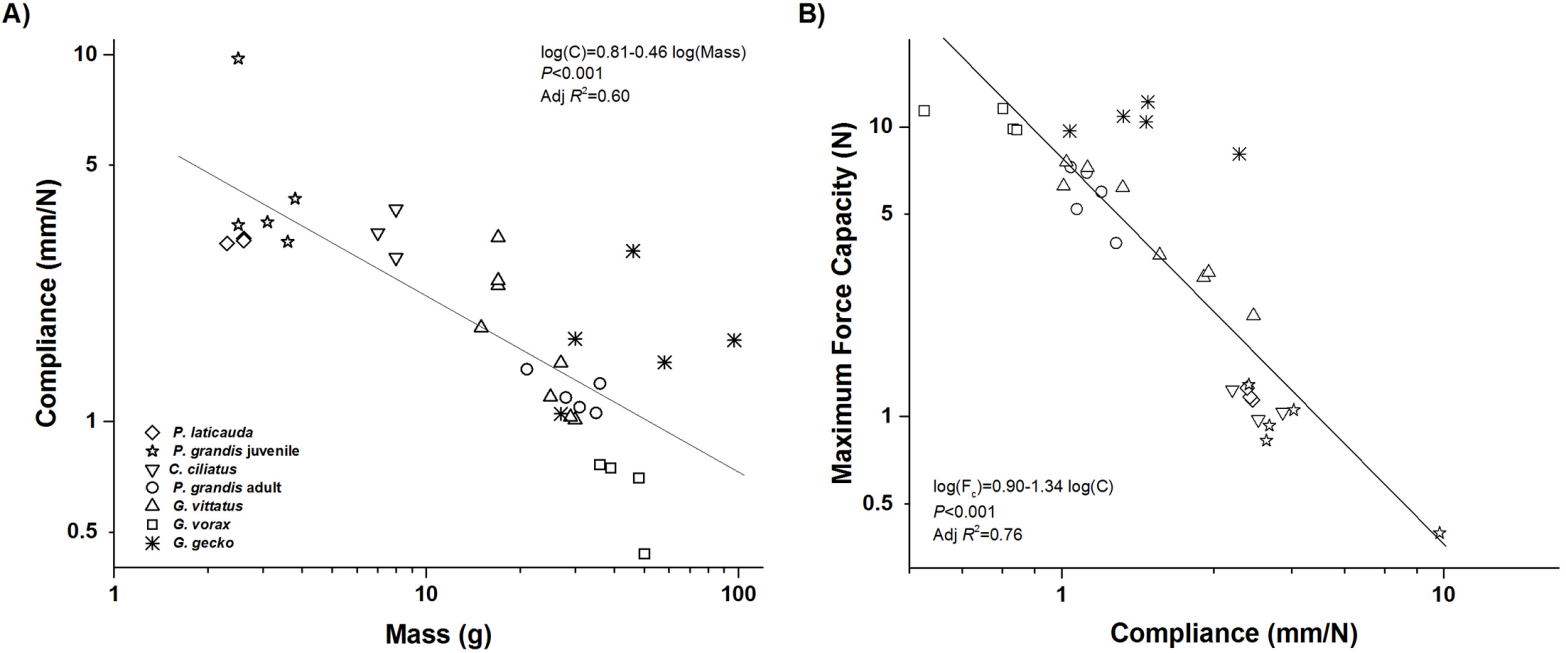
Relationship between system (animal) compliance, animal size, and maximum adhesive force. A) Gecko compliance significantly decreases as animal size increases. B) Maximum adhesive force significantly decreases with increasing gecko compliance (as animals get smaller).

As predicted, the gecko adhesive system becomes less compliant (stiffer) as geckos become larger. There was a significant negative relationship between system compliance and body size across gecko species, although there remains a substantial amount of unexplained variation (log(*C*) = 0.81–0.46log(Mass), *P*<0.001, Adj. *R* ^2^ = 0.60, Fig 4A), (log(*C*) = 2.78–1.29log(SVL), *P*<0.001, Adj. *R* ^2^ = 0.58). Further, we also found a significant negative relationship between maximum adhesive force and compliance, with smaller geckos showing a more compliant adhesive system than larger geckos (log(*F*_*c*_) = 0.90–1.34log(*C*), *P*<0.001, Adj. *R* ^2^ = 0.76, Fig 4B).

**Fig 5:**
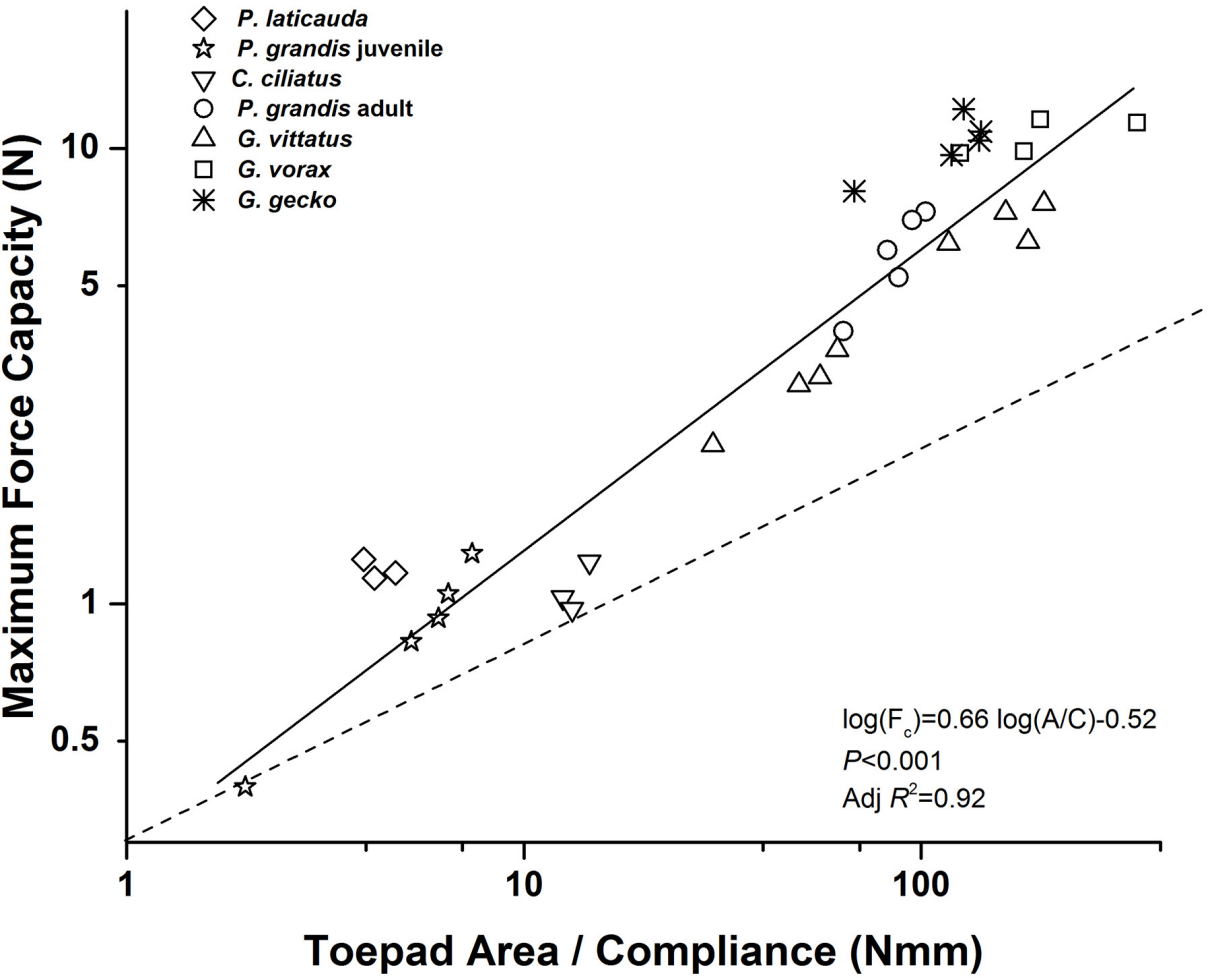
Relationship between maximum adhesive force and the area to compliance ratio. There is a significant relationship between maximum adhesive force and the area to compliance ratio, with little unexplained variability (~ 8%), which shows that it is the combination of the gecko’s compliance and adhesive toepad area that best explains variation in maximum adhesive force. Note: Dotted line shows slope of 0.5, for reference.

Lastly, we found that the best predictor of maximum adhesive force in geckos across the six species we tested was the ratio of toepad area to compliance (*A/C)*, (log(*F*_*c*_) = 0.66log(*A*/*C*)– 0.52, *P*<0.001, Adj. *R* ^2^ = 0.92, Fig 5). Indeed, this ratio explained 92% of the variation in maximum adhesive force, compared to 78% when only toepad area was included in the model. Thus, the addition of compliance as a variable explained approximately an additional 14% of variation in maximum adhesive force.

Relationships between variables within species were similar to relationships across species. There was a significant and positive relationship between body mass and maximum adhesive force (log(*F*_*c*_) = 0.86log(Mass)– 0.50, *P*<0.001, Adj. *R* ^2^ = 0.94) and mass and toepad area within *P*. *grandis* (log(*A*) = 0.68log(Mass) + 1.00, *P*<0.001, Adj. *R* ^2^ = 0.99). Maximum adhesive force increased significantly with increases in toepad area (log(*F*_*c*_) = 0.96log(*A*)– 1.75, *P*<0.001, Adj. *R* ^2^ = 0.93). There was a significant negative relationship between system compliance and body size (log(*C*) = 0.91–0.57log(Mass), *P*<0.001, Adj. *R* ^2^ = 0.83) and between maximum adhesive force and compliance within this species (log(*F*_*c*_) = 0.83–1.39log(*C*), *P*<0.001, Adj. *R* ^2^ = 0.95). Similar to our across-species results, we found that the best predictor of maximum adhesive force was the ratio of toepad area to compliance (*A/C)*, (log(*F*_*c*_) = 0.69log(*A*/*C*)– 0.56, *P*<0.001, Adj. *R* ^2^ = 0.99). There were no significant differences in slope between the regression models produced using all of the species versus just the *P*. *grandis* (t-tests: *Fc* vs. Mass, *t*_39_ = 0.06, *P* = 0.95; *A* vs. Mass, *t*_39_ = 2.35, *P* = 0.02; *Fc* vs. *A*, *t*_39_ = 2.05, *P* = 0.05; *C* vs. Mass, *t*_39_ = 1.01, *P* = 0.32; *Fc* vs. *C*, *t*_39_ = 0.30, *P* = 0.77; *Fc* vs. *A*/*C*, *t*_39_ = 0.74, *P* = 0.46; all non-significant with sequential Bonferroni test).

**Fig 6:**
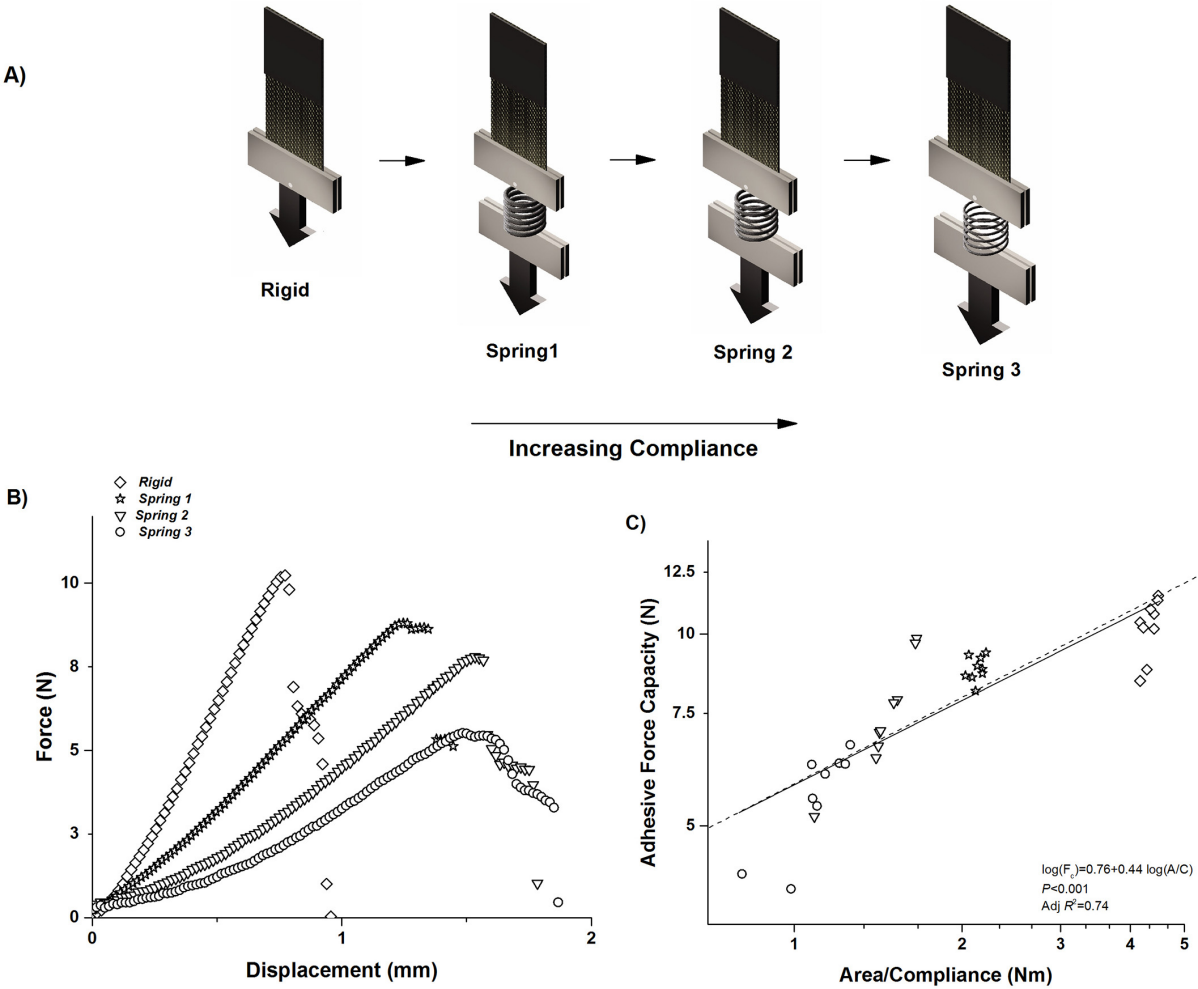
Results from tests of system compliance using a range of spring compliance. A) Experimental set up of “model gecko”. A gecko-like adhesive created in the same manner described in prior publications [25], namely stiff fabric with a coating of a soft elastomer (see materials and methods for details), is attached to a glass plate fixed in the Instron in the same manner as in geckos, springs of variable compliance mimic whole body compliance found in natural gecko species. B) Force-extension output of four springs, with the adhesive pad area (A = 300 mm^2^) remaining constant for all experiments. Increasing slope with increased spring stiffness mimics results found in geckos. C) Scaling relationship of maximum adhesive force versus area to compliance ratio. Note, that $C_{\;spring\;3}>C_{\;spring\;2}>C_{\;spring\;1}{}_{}>C_{\;Rigid}$.

Our synthetic pad experiments (Fig 6A) support the above findings. We observed that as the compliance of the synthetic tendon (i.e. spring) increased, maximum adhesive force decreased (Fig 6B), and these data points continue to follow the *A/C* ratio (Fig 6C). By performing these measurements with systems comprised of springs with different compliances, the value of maximum adhesive force decreases as *C* increases. Importantly, in these experiments, nothing changes with regard to the pad (such as *A or G*_*c*_) or glass substrate; only the system compliance is altered. *G*_*c*_ is the energy per unit area defined by the nature of the bonds at the interface [24–25].

## Discussion

Our data provide strong evidence for our hypothesis that changes in compliance as climbing animals change in size is a major contributor to the scaling of adhesion. The addition of system compliance along with toepad area explains 92% of the variation in maximum adhesive force among 6 gecko species. These data thus indicate that the adhesive system of larger geckos is stiffer than for smaller geckos, and this increased stiffness enables larger geckos to increase their adhesive abilities to enable them to climb. This result is novel because it reveals how differences in basic material properties and geometry of living systems enable them to obtain new adhesive properties. Additionally, our results suggest that the emphasis on area as the key parameter controlling the scaling of adhesion is incomplete. We caution that our sample is a small portion of overall gecko diversity (>800 species [5]), and therefore our results require additional verification.

Climbing in geckos is complex, and maximum adhesive force as measured here does not fully explain or predict every aspect of gecko adhesion in terms of their ability to climb. The additional remaining 8% of variation could be attributed to many factors, including measurement error, behavioral variation, or other unaccounted-for material properties, and deserves further research. However, our scaling relationship demonstrates how adhesive force can be generated in the gecko adhesive system and the maximum force can provide an upper bound on the mass or force that a gecko can support while hanging and climbing. Although this force is many times greater than the gecko’s mass, the forces generated during dynamic events such as rapid accelerations during climbing, jumping and reattaching during a fall are much greater than the gecko’s mass [14]. The ability to accomplish these dynamic events contributes to overall fitness and thus measuring maximum adhesive force can provide an upper bound for gecko mass even if typical climbing forces or hanging masses are less than the maximum forces measured in our experiments.

Our synthetic pad experiments also demonstrate that to scale maximum adhesive force, structures beyond the interface play an important, potentially primary role. Thus, the maintenance of stiffness in the loading direction from the interface to the load bearing skeleton is critical for scaling maximum adhesive force and must be balanced in a non-trivial manner with compliance of the attachment pad perpendicular to the surface. One difference between the synthetic pad experiments and the live gecko tests is that geckos possess several toes that vary in angle when climbing, or when being used in maximum adhesion tests. We tested for the effects of toe angle by creating a three “toed” set of synthetic pads connected to a larger “foot” (S2 and S3 Figs), and varied “toe” angles between 0–45 degrees at 15 degree intervals (S3 Fig), and found that varying the angle of these synthetic “toes” had no significant impact on the basic relationship between maximum adhesive force and A/C (S4–S6 Figs). We also examined toe angles for the front feet of four *Gehyra vorax* when they were adhering on the instron at peak forces, and found that the average angle among all toes was 15.4±0.4 (SE) degrees, which is within this experimental range. This finding supports the claim that the system compliance [36,37] in the direction of loading is a quantitative parameter that accounts for both materials properties and complex geometric arrangements of mechanical elements and is an important quantity in understanding the development of maximum adhesive force.

Whereas our results indicate an important role for compliance for controlling maximum adhesive force in geckos, we have not addressed potential mechanisms by which geckos can alter compliance, but we mention several possibilities below. Compliance, in the simplest sense, is inversely related to a structure’s cross-sectional size and the material’s elastic moduli. In a system as complex as a gecko, many structures connect to each other, and system compliance is defined by the sum of the compliances for structures that are connected in series (toepad, limb, body). Therefore, a weak, or more compliant, linking structure will significantly impact the full system compliance, as demonstrated in our “model gecko” synthetic adhesive experiments. To minimize system compliance, geckos have developed unique integrated structures of stiff tissues, namely tendons that integrate into the skin within the toepad.

Indeed, detailed anatomical studies [10], primarily of the tokay gecko (*Gekko gecko*) show that a stiff loading path exists in geckos which connects the setae to the rigid skeletal system via a unique tendon arrangement relative to other lizards. At the substrate interface, the terminal fibrillar features are made of stiff keratin fibers, which connect directly to a thin epidermal layer. The epidermal layer is rigidly bound to the lateral digital tendon through an interdigitated connection with the stratum compactum of the dermis. Finally, the tendon then connects directly to the skeletal system of the gecko through the metacarpo- or metatarso-phalangeal joint capsule [20]. Interestingly this system of connections is only seen near the setae on the ventral side of the toe, and is absent in the dorsal region, where compliant tissues and connections are observed [20]. However, gecko species vary in tendon anatomy [5], with some species exhibiting highly complex tendon structure, and other species displaying more simplified anatomical arrangements. Moreover, no studies have examined the relative dimensions of these different arrangements, nor their material properties. Such data on how tendon size and resultant material properties change among geckos of different body sizes could be informative for understanding how compliance in geckos changes with size. We also note that additional data on the habitat use (e.g., substrate use) of different gecko species, especially in relation to their tendon anatomy, body size, and overall adhesive capacities, would be valuable for determining whether species can specialize on individual surfaces. While our sample is too modest to do so, a more extensive phylogenetic analysis of these factors using established phylogenetic methods would be valuable.

In this paper, we have not quantified the material properties of individual tissues among different gecko species, although we are actively investigating this issue (Kuo et al., unpub. data). These data will enable us to test whether material properties of tendons and other tissues change among gecko species, or within species of different sizes. Further, these data will clarify whether changes in compliance with size are a function of differential structural arrangements among different species. It is important to note that the tendons are not the only contributors to the overall compliance of the gecko adhesive system and anatomical work that examines material properties of other elements, such as other soft tissue, muscle, and bone, and modelling studies that provide an integrative model for how these different elements contribute to overall system compliance would be valuable.

In conclusion, our data provide a more holistic view of adhesion in climbing animals, and opens the door to more integrative studies of material properties of both setae and other anatomical elements in the toepad, limb, and body. We note that our modest sample requires more data from other researchers to test our findings before any strong generalizations can be made. Our hope is that this integrative approach will allow us to more fully understand the evolution of climbing structures.

## Supporting Information

S1 Fig Force-extension plots. Plots of force and extension for lap shear tests of geckos/synthetic adhesives on glass. A) *Gehyra vorax* on glass at 300 mm/min. B) synthetic adhesives on glass at 10 mm/min. The two data points highlighted in each figure correspond to transitions in the shear adhesion measurements. The first datum (I.) in S1A Fig, is the point at which a linear relationship begins, corresponding qualitatively with the instance when the specimen’s forearms were completely extended. In S1B Fig, the first datum (I.) is the initial minimum force before loading the adhesive. The second datum (II.) is the initial drop in force, corresponding to the onset of slip between the specimen toepad or adhesive pad and the glass substrate. The force for this second datum is the maximum force capacity (*F*_*c*_) for the gecko, and maximum adhesive force capacity for the synthetic adhesive. A least squares linear fit was applied to the data between the first and second data points. The slope of the least squares fit is the system stiffness, from which the system compliance (*C*) is calculated as the inverse.
(TIF)

S2 Fig Synthetic gecko toe geometry. Geometry of a single synthetic adhesive digit.
(TIF)

S3 Fig Digit testing setup and schematic. a) Picture of testing setup. Synthetic adhesive digits are attached to 3D printed “wrist” via a screw through a hole in the polycarbonate grips–allowing rotational freedom. The wrist is then attached to an aluminum anchor, which is itself attached to the Instron testing machine. The adhesive pads are pressed into contact with a glass plate that is attached to a 2kN load cell. b) Schematic of relevant testing geometry. A ruler was used to make marks on the wrist of our setup (a) indicating the angle of an outer digit with respect to the center digits. Both the wrist and the center digit were aligned parallel with the displacement direction.
(TIF)

S4 Fig Variable angle digit testing setup and schematic. a) Picture of testing setup. Synthetic adhesive digits anchored at one end to the wrist are adhered to glass at different angles with respect to the center digit and wrist. b) Schematic of relevant testing geometry. A ruler was used to make marks on the wrist of our setup (a) indicating the angle of an outer digit with respect to the center digits. Both the wrist and the center digit were aligned parallel with the displacement direction. The outer digits vary from 0° to 45° with respect to the displacement direction.
(TIF)

S5 Fig Force capacity versus digit angle. Force Capacity and Compliance vs. Outer Digit Angle. Based on a one-way ANOVA, followed by post-hoc Tukey’s HSD pair-wise comparisons, different letters indicate significantly different values. Same letters indicate non-significant values.
(TIF)

S6 Fig Adhesive Force Capacity vs. Area/Compliance. The data from the current tests, as well as the tests quoted in the original manuscript are plotted.
(TIF)

S1 File Experiments with synthetic three “toed” gecko system. (DOCX)

## References

[1] Irschick DJ, Austin CC, Petren K, Fisher RN, Losos JB, Ellers O. A comparative analysis of clinging ability among pad-bearing lizards. Biol. J. Linn. Soc. 1996; 59: 21–35.

[2] Gorb SN. The design of the fly adhesive pad: distal tenent setae are adapted to the delivery of an adhesive secretion. Proc. Royal Soc. London B. B 1998; 265:747–752.

[3] Federle W, Barnes WJP, Baumgartner W, Drechsler P, Smith JM. Wet but not slippery: Boundary friction in tree frog adhesive toe pads. J. Royal Soc. Int. 2006; 3: 689–697. 10.1098/rsif.2006.0135 PMC1664653 16971337

[4] Creton C, Gorb SN. Sticky feet: from animals to materials. MRS Bull. 2007; 32: 466–468.

[5] Gamble T, Greenbaum E, Jackman TR, Russell AP, Bauer AM. Repeated Origin and Loss of Adhesive Toepads in Geckos. Plos One 2012; 7: e39429 10.1371/journal.pone.0039429 22761794 PMC3384654

[6] Lee H, Lee BP, Messersmith PB. A reversible wet/dry adhesive inspired by mussels and geckos. Nature 2007; 448: 338–41. 17637666 10.1038/nature05968

[7] Williams EE, Peterson JA. Convergent and alternative designs in the digital adhesive pads of scincid lizards. Science 1982; 215: 1509–1511. 17788677 10.1126/science.215.4539.1509

[8] Autumn K, Sitti M, Liang YA, Peattie AM, Hansen WR, Sponberg S et al Evidence for van der Waals adhesion in gecko setae. Proc. Nat. Acad. Sci. 2002; 99: 12252–12256. 12198184 10.1073/pnas.192252799 PMC129431

[9] Ernst VV, Ruibal R. The structure and development of the digital lamellae of lizards. J. Morph. 1967; 120: 233–266. 10.1002/jmor.1051200303 5970300

[10] Russell AP. The morphological basis of weight bearing in the scansors of the Tokay Gecko (Reptilia:Sauria). Can. J. Zool. 1986; 64: 948–955.

[11] Bauer AM, Russell AP. Morphology of gekkonoid cutaneous sensilla, with comments on function and phylogeny in the Carphodactylini (Reptilia: Gekkonidae). Can. J. Zool. 1988; 66: 1583–1588.

[12] Huber G, Gorb SN, Spolenak R, Arzt E. Resolving the nanoscale adhesion of individual gecko spatulae by atomic force microscopy. Bio. Lett. 2005; 1: 2–4. 17148113 10.1098/rsbl.2004.0254 PMC1629055

[13] Autumn K, Liang YA, Hsieh ST, Zesch W, Chan WP. Adhesive force of a single gecko foot-hair. Nature 2000; 405: 681–685. 10864324 10.1038/35015073

[14] Autumn K. How gecko toes stick—The powerful, fantastic adhesive used by geckos is made of nanoscale hairs that engage tiny forces, inspiring envy among human imitators. Am. Sci. 2006; 94: 124–132.

[15] Webster NB, Johnson MK, Russell AP. Ontogenetic scaling of scansorial surface area and setal dimensions of *Chondrodactylus bibronii* (Gekkota: Gekkonidae): testing predictions derived from cross-species comparisons of gekkotans. Acta Zool. 2009; 90: 18–29

[16] Russell AP, Johnson MK. Between a rock and a soft place: microtopography of the locomotor substrate and the morphology of the setal fields of Namibian day geckos (Gekkota: Gekkonidae: *Rhoptropus*). Acta Zoologica 2014; 95: 299–318.

[17] Bauer AM, Good DA. Scaling of scansorial surface area in the genus *Gekko* In: Rocek Z, editor. Studies in herpetology. Charles University; 1986 pp. 363–366.

[18] Elstrott J, Irschick DJ. Evolutionary correlations among morphology, habitat use and clinging performance in Caribbean *Anolis* lizards. Biol. J. Linn. Soc. 2004; 83: 389–398.

[19] Irschick DJ, VanHooydonck B, Meyers J, Herrel A. Intraspecific correlations among morphology, performance, and habitat use within a green anole lizard (*Anolis carolinensis*) population. Biol. J. Linn. Soc. 2005; 85: 211–221.

[20] Russell AP. Integrative functional morphology of the gekkotan adhesive system (Reptilia: Gekkota). Int. Comp. Biol. 2002; 42: 1154–1163. 10.1093/icb/42.6.1154 21680400

[21] Scherge M, Gorb S. Biological micro- and nanotribology: Nature’s solutions Springer Press, 306 pp; 2001.

[22] Arzt E, Gorb S, Spolenak R. From micro to nano contacts in biological attachment devices. Proc Nat Acad Sci. 2003; 100: 10603–10606. 12960386 10.1073/pnas.1534701100 PMC196850

[23] Peattie AM, Full RJ. Phylogenetic analysis of the scaling of wet and dry biological fibrillar adhesives. Proc. Nat. Acad. Sci. 2007; 104: 18595–18600. 18000044 10.1073/pnas.0707591104 PMC2141822

[24] Bartlett MD, Croll AB, Crosby AJ. Designing bio-inspired adhesives for shear loading: From simple structures to complex patterns. Adv. Funct. Mat. 2012a; 22: 4985–4992.

[25] Bartlett MD, Croll AB, King DR, Paret BM, Irschick DJ, Crosby AJ. Looking beyond fibrillar features to scale gecko-like adhesion. Adv. Mat. 2012b; 24: 1078–1083. 10.1002/adma.201104191 22278804

[26] Bartlett MD, Crosby AJ. Scaling normal adhesion force capacity with a generalized parameter. Langmuir 2013; 29: 11022–11027. 10.1021/la4013526 23924148

[27] King DR, Bartlett MD, Gilman CA, Irschick DJ, Crosby AJ. Creating gecko-like adhesives for “real world” surfaces. Adv. Mat. 2014; 26: 4345–4351. 10.1002/adma.201306259 24740961

[28] Russell AP. Some comments regarding interrelationships among gekkonine geckos In: Bellairs A, Cox DB, editors. Morphology and Biology of Reptiles, Linnean Society Symposium. Academic Press; 1976 Series 3. pp. 217–244.

[29] Irschick DJ, Herrel A, VanHooydonck B. Whole-organism studies of adhesion in pad-bearing lizards: Creative evolutionary solutions to functional problems. J. Comp. Phys. A. 2006; 192: 1169–1177. 10.1007/s00359-006-0145-2 16957944

[30] Autumn K, Gravish N. Gecko adhesion: evolutionary nanotechnology. Phil. Trans. Royal. Soc. London 2008; 366: 1575–1590. 10.1098/rsta.2007.2173 18192170

[31] Jeong HE, Suh KY. Nanohairs and nanotubes: Efficient structural elements for gecko-inspired artificial dry adhesives. Nano Today 2009; 4: 335–346.

[32] Sameoto D, Menon C. Recent advances in the fabrication and adhesion testing of biomimetic dry adhesives. Smart Mat. and struct. 2010; 19: 103001.

[33] Gillies AG, Henry A, Lin H, Shiuan RS, Fearing RS, Full RJ. Gecko toe and lamellar shear adhesion on macroscopic engineered rough surfaces. J. Exp. Biol. 2014; 217: 283–289. 10.1242/jeb.092015 24115057

[34] Bergmann PJ, Irschick DJ. Effects of temperature on maximum clinging ability in a diurnal gecko: evidence for a passive clinging mechanism? J. Exp. Zool. A. 2005; 303: 785–791. 10.1002/jez.a.210 16106405

[35] Meiri S. Length–weight allometries in lizards. J. Zool. 2010; 281: 218–226.

[36] Barquins M. Influence of the stiffness of testing machines on the adherence of elastomer. J. App. Polm. Sci. 1983; 28: 2647.

[37] Johnson KL. Contact Mechanics. Cambridge University Press, 448 pp; 1985.

