# Supplementary figures and images for: Geckos as Springs: Mechanics Explain Across-Species Scaling of Adhesion
Source: PLoS One. 2015 Sep 2;10(9):e0134604. doi: 10.1371/journal.pone.0134604 (PMC4558017; doi:10.1371/journal.pone.0134604)

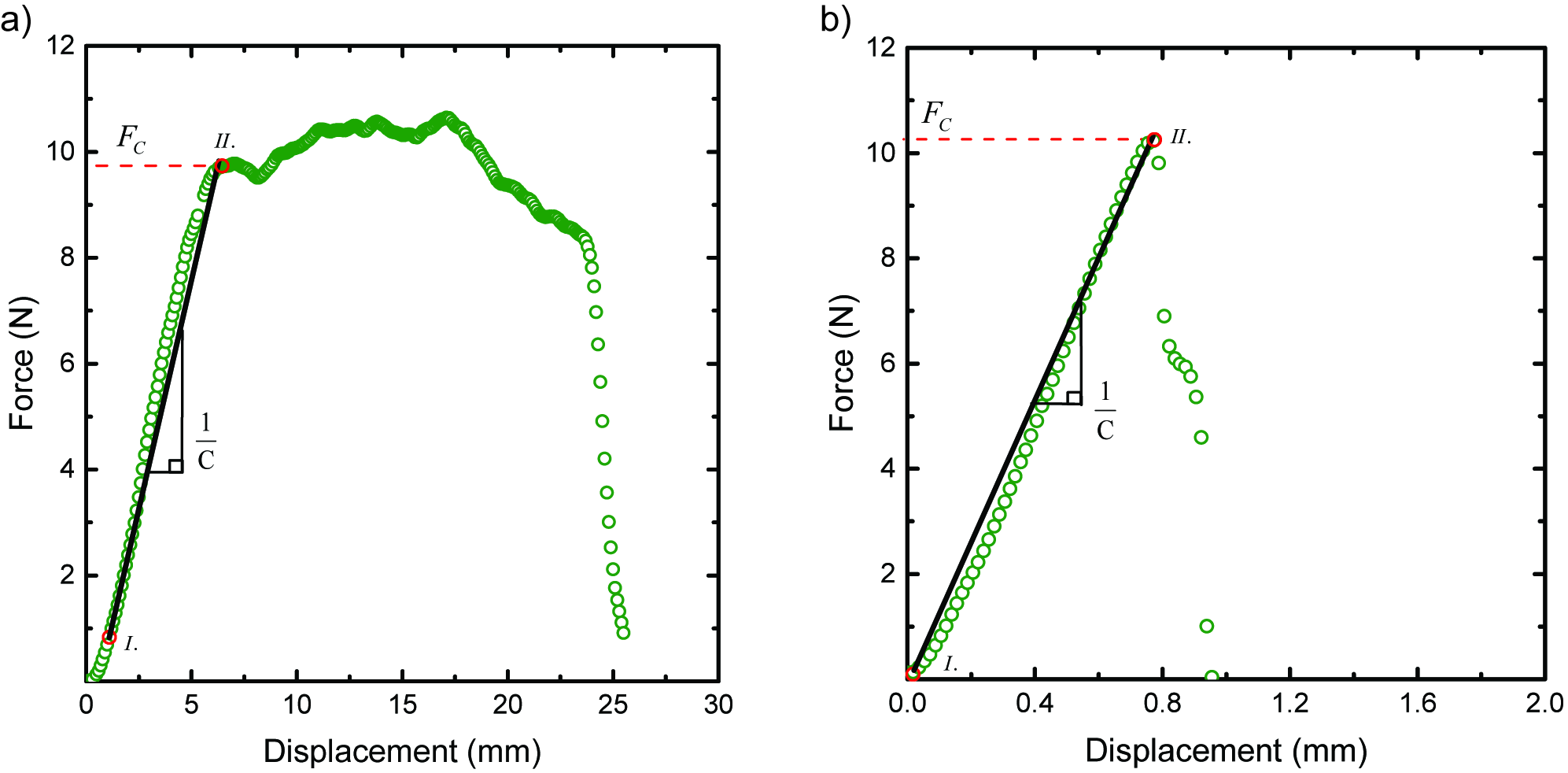

Supplement: S1 Fig — Plots of force and extension for lap shear tests of geckos/synthetic adhesives on glass. A) Gehyra vorax on glass at 300 mm/min. B) synthetic adhesives on glass at 10 mm/min. The two data points highlighted in each figure correspond to transitions in the shear adhesion measurements. The first datum (I.) in S1A Fig, is the point at which a linear relationship begins, corresponding qualitatively with the instance when the specimen’s forearms were completely extended. In S1B Fig, the first datum (I.) is the initial minimum force before loading the adhesive. The second datum (II.) is the initial drop in force, corresponding to the onset of slip between the specimen toepad or adhesive pad and the glass substrate. The force for this second datum is the maximum force capacity (F c) for the gecko, and maximum adhesive force capacity for the synthetic adhesive. A least squares linear fit was applied to the data between the first and second data points. The slope of the least squares fit is the system stiffness, from which the system compliance (C) is calculated as the inverse. (TIF) [file pone.0134604.s001.tif]

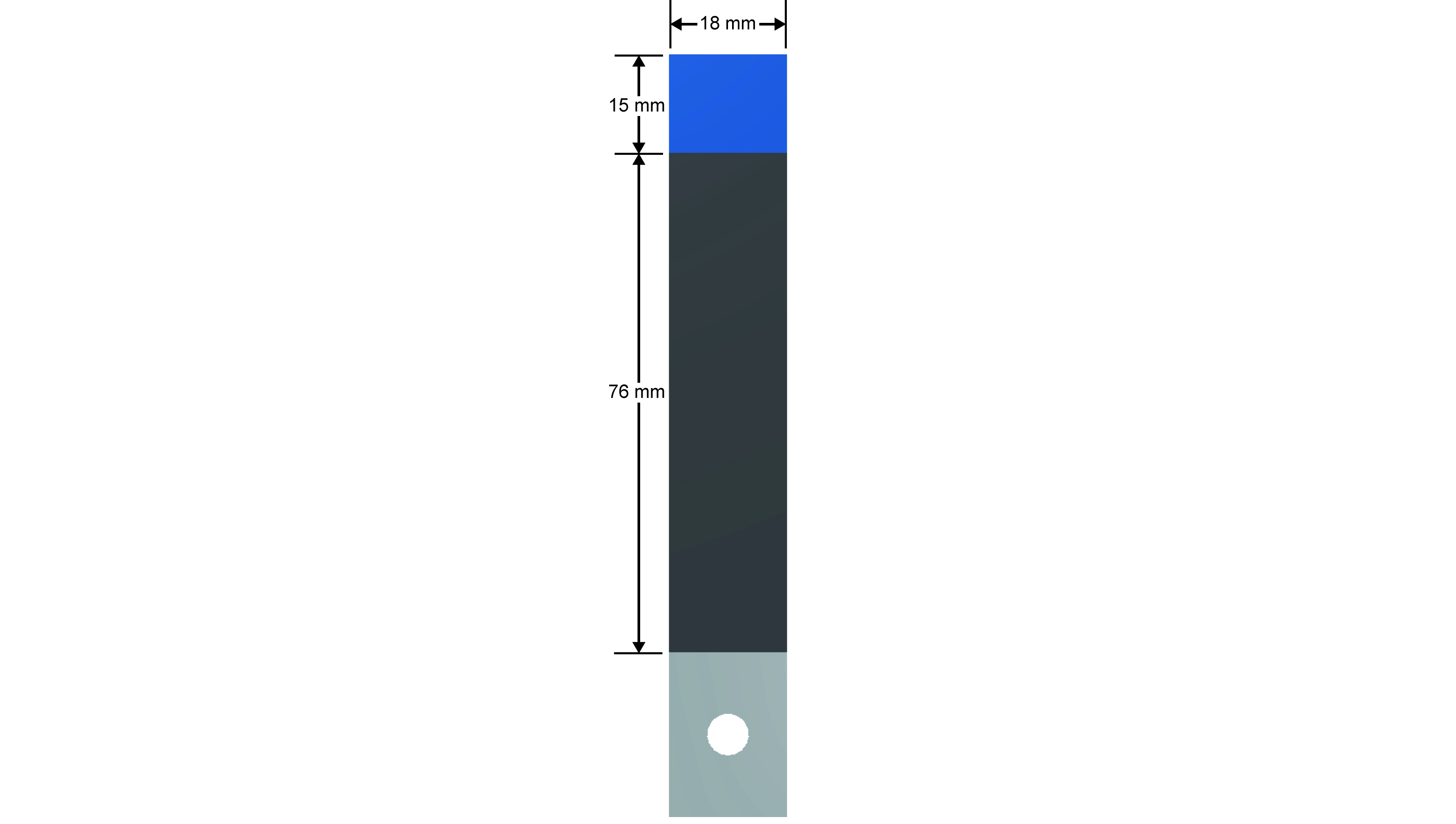

Supplement: S2 Fig — Geometry of a single synthetic adhesive digit. (TIF) [file pone.0134604.s002.tif]

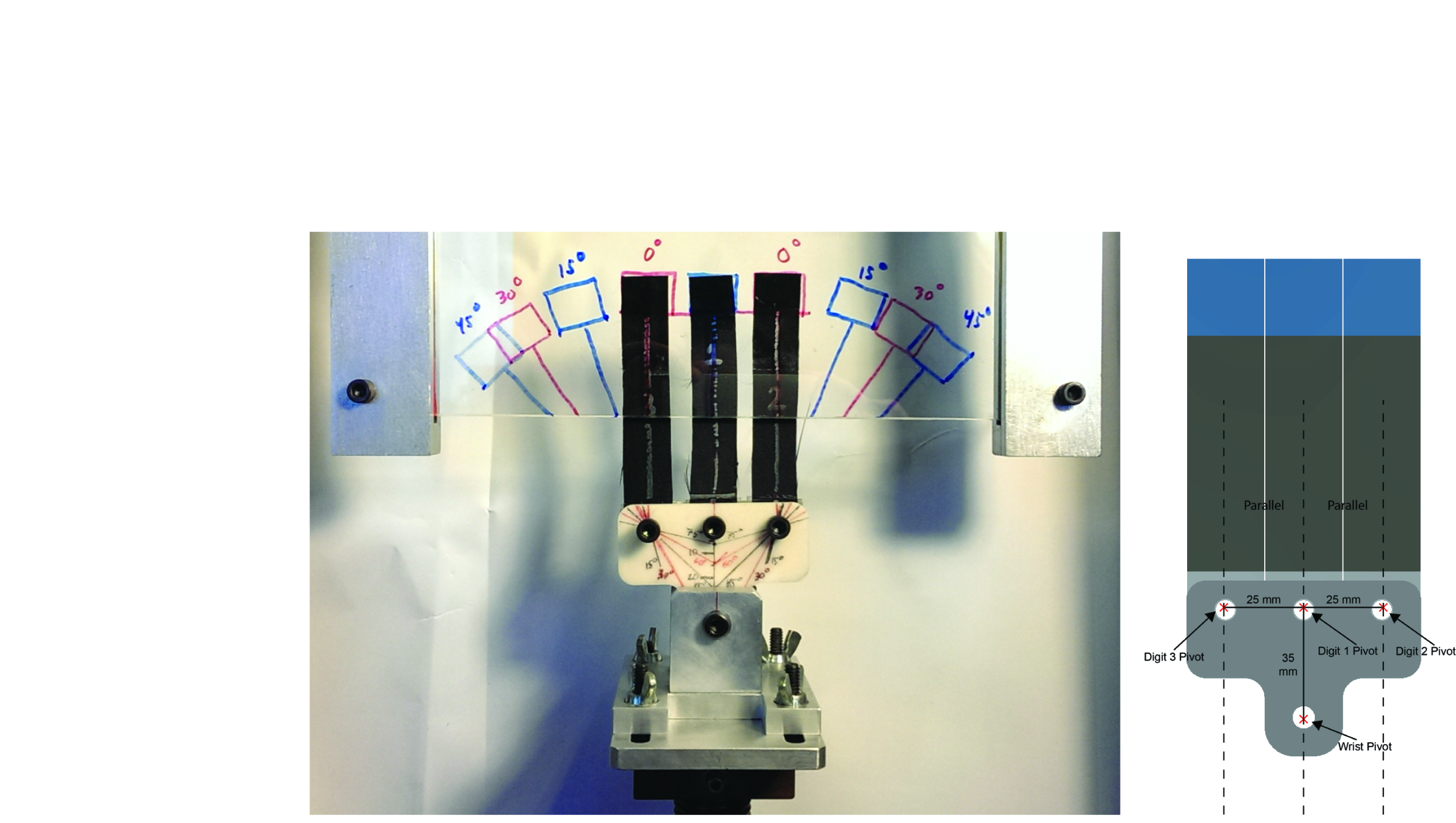

Supplement: S3 Fig — a) Picture of testing setup. Synthetic adhesive digits are attached to 3D printed “wrist” via a screw through a hole in the polycarbonate grips–allowing rotational freedom. The wrist is then attached to an aluminum anchor, which is itself attached to the Instron testing machine. The adhesive pads are pressed into contact with a glass plate that is attached to a 2kN load cell. b) Schematic of relevant testing geometry. A ruler was used to make marks on the wrist of our setup (a) indicating the angle of an outer digit with respect to the center digits. Both the wrist and the center digit were aligned parallel with the displacement direction. (TIF) [file pone.0134604.s003.tif]

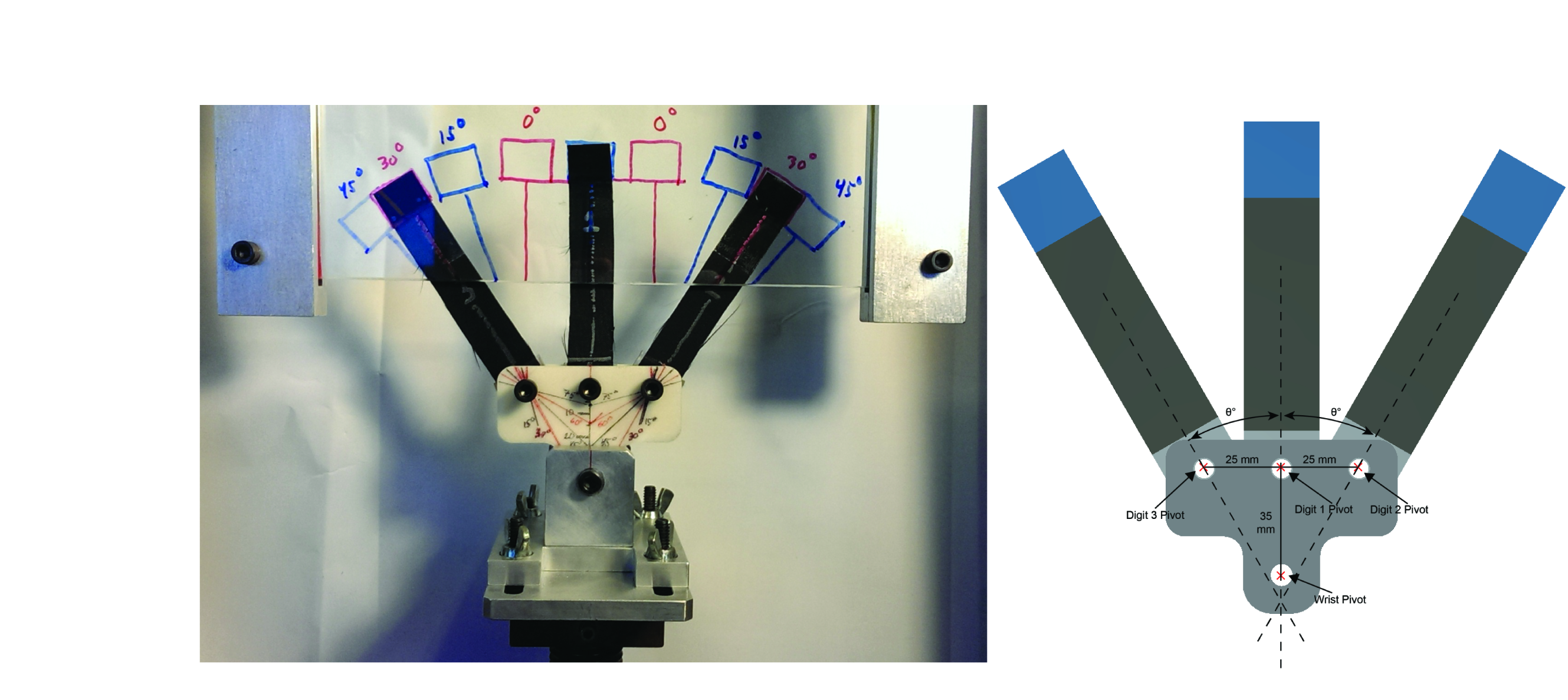

Supplement: S4 Fig — a) Picture of testing setup. Synthetic adhesive digits anchored at one end to the wrist are adhered to glass at different angles with respect to the center digit and wrist. b) Schematic of relevant testing geometry. A ruler was used to make marks on the wrist of our setup (a) indicating the angle of an outer digit with respect to the center digits. Both the wrist and the center digit were aligned parallel with the displacement direction. The outer digits vary from 0° to 45° with respect to the displacement direction. (TIF) [file pone.0134604.s004.tif]

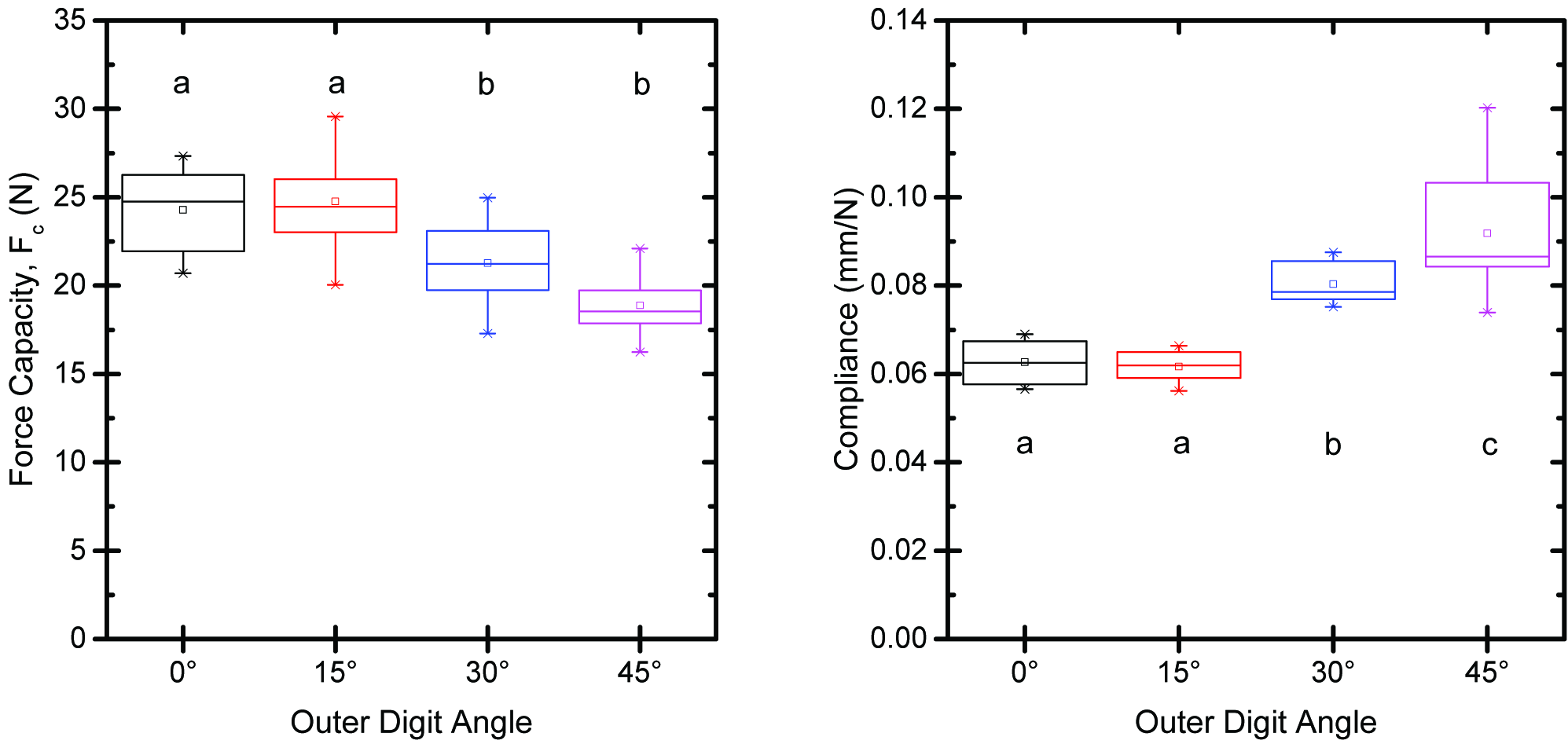

Supplement: S5 Fig — Force Capacity and Compliance vs. Outer Digit Angle. Based on a one-way ANOVA, followed by post-hoc Tukey’s HSD pair-wise comparisons, different letters indicate significantly different values. Same letters indicate non-significant values. (TIF) [file pone.0134604.s005.tif]
