# Supplementary material for: Geckos as Springs: Mechanics Explain Across-Species Scaling of Adhesion
Source: PLoS One. 2015 Sep 2;10(9):e0134604. doi: 10.1371/journal.pone.0134604 (PMC4558017; doi:10.1371/journal.pone.0134604)
Supplement: S1 File — (DOCX) [file pone.0134604.s007.docx]

**Supporting information**

**Experiments with synthetic three “toed” gecko system**

To demonstrate the generality of the scaling relationship between force capacity and the ratio of area and compliance, we measured the force capacity of integrated systems of adhesive samples. Each integrated system included adhesive samples, or digits, connected to a common, rigid mount, which we refer to as a wrist. The connection between each digit and the wrist allows for rotational freedom. The adhesive digits were comprised of a nylon fabric and a crosslinked polydimethylsiloxane (PDMS) elastomer (Dow Corning’s Sylgard 184). The nylon was purchased from JoAnn Fabrics with a weight of 95 g/m^2^. The PDMS was received from Dow-Corning and mixed in a prepolymer:crosslinker ratio of 10:1. Mixed and degassed PDMS was poured into a 1.5 mm glass, and the nylon fabric was placed on top. The PDMS-fabric composite was cured at room temperature for 18 hours and then in an oven at 70°C for 2 hours. Each adhesive digit was cut from a larger sample, such that the individual samples were 15 mm in width and 18 mm in pad length. Polycarbonate panels were super-glued to the bottom of the fabric to provide a gripping point for testing. A schematic of a synthetic adhesive is shown in S2 Fig. To measure the force capacity of this integrated system, the digits were anchored to the wrist, which was anchored to the bottom fixture of an Instron testing machine (Model 5564), as seen in S3 Fig. The PDMS pads of each digit were then pressed into contact with a glass plate, which was held in a custom-built aluminum frame anchored to the top fixture of the Instron. We performed tests where the angle of the outer digits was changed with respect to the center digit and the wrist. A schematic of a test is shown in S4 Fig.

The displacement rate of the Instron fixture was 10 mm/min. One set of tests was comprised of a single digit in the center, oriented parallel to the testing direction. Each of the three selected synthetic adhesive digits were tested 10 times. Another set of tests consisted of three digits attached to the wrist, where the middle digit was oriented in the direction of testing, and the angle of the outer digits with respect to the center digit varied from 0° to 45°. Each angular configuration was tested 10 times.

Toe Angle during Maximum Force Capacity

We tested a subset of the larger lizards in random order, on the same day (*Gehyra vorax*, *N*=4), and took images of the front feet during the point of maximum force capacity in these trials to measure the angles between toes later, using ImageJ (Rasband, 1997–2015).

**Results**

From the measured force versus displacement data, we can determine the maximum adhesive force (F_c_) and the system compliance during loading (C), in the same manner as mentioned in the Methods section in the main text. Plots of F_c_ and C vs. outer digit angle are shown in S3 Fig. To test for differences in F_c_ with outer digit angle, we conducted a one-way ANOVA, followed by post-hoc Tukey’s HSD pair-wise comparisons between angular configurations (S5 Fig.). F_c_ is plotted against A/C, as shown in S6 Fig. A power law fit was applied to the as measured data using a damped least-squares method (Levenberg-Marquardt). The power law scaling was determined to be 0.546 with an R_­_^2^ = 0.93. This is plotted on a log-log scale in S6 Fig.

Toe angles during maximum force capacity measurements ranged from a mean of 13.5 to 17.7 degrees per individual, between all toes (combined-animal mean: 15.4 ± 1.0 for 5-8 measurements of maximum force capacity per individual).

**Discussion of results**

Our measurements indicate that there is no significant difference in both F_c_ and C for the testing of devices which have relative angles between 0° and 15° between the center and outer digits. However, when the relative angle increased to between 30° and 45°, the data indicates a significant difference in both F_c_ and C between 0° and the higher angles. This change in C, and associated change in Fc, is consistent with expected system mechanics and the predictions of the scaling relationship for Fc. Importantly, when F_c_ is compared to A/C, a 0.546 dependence of F_c_ on A/C is determined, which compares favorably to the predicted scaling of 0.5, as well as with the gecko testing results (0.62). As predicted from the derivation of the scaling relationship, F_c_ is expected to scale with the ratio of pad area (A), and full system compliance, C. The arrangement of the digits, similar to the complex arrangements of limbs and digits in a gecko, is a geometric factor in determining the full system compliance. Hence the arrangement of the digits only affects the magnitude of C and F_c_, but not the relationship between F_c_ and the ratio of A/C.
